# Comparative genome and phenotypic analysis of three *Clostridioides difficile* strains isolated from a single patient provide insight into multiple infection of *C. difficile*

**DOI:** 10.1186/s12864-017-4368-0

**Published:** 2018-01-02

**Authors:** Uwe Groß, Elzbieta Brzuszkiewicz, Katrin Gunka, Jessica Starke, Thomas Riedel, Boyke Bunk, Cathrin Spröer, Daniela Wetzel, Anja Poehlein, Cynthia Chibani, Wolfgang Bohne, Jörg Overmann, Ortrud Zimmermann, Rolf Daniel, Heiko Liesegang

**Affiliations:** 1 Institute for Medical Microbiology, University Medical Center Göttingen, Göttingen, Germany; 2 Department of Genomic and Applied Microbiology & Göttingen Genomics Laboratory, Institute of Microbiology and Genetics, Georg-August-University, Göttingen, Germany; 3 Leibniz Institute DSMZ-German Collection of Microorganisms and Cell Cultures, Braunschweig, Germany; 4 German Center for Infection Research (DZIF), Partner Site Hannover-Braunschweig, Braunschweig, Germany

**Keywords:** *Clostridioides difficile*, *Clostridium difficile*, Multiple infection, Genetic switch, Genomic adaptation, Horizontal gene transfer

## Abstract

**Background:**

*Clostridioides difficile* infections (CDI) have emerged over the past decade causing symptoms that range from mild, antibiotic-associated diarrhea (AAD) to life-threatening toxic megacolon. In this study, we describe a multiple and isochronal (mixed) CDI caused by the isolates DSM 27638, DSM 27639 and DSM 27640 that already initially showed different morphotypes on solid media.

**Results:**

The three isolates belonging to the ribotypes (RT) 012 (DSM 27639) and 027 (DSM 27638 and DSM 27640) were phenotypically characterized and high quality closed genome sequences were generated. The genomes were compared with seven reference strains including three strains of the RT 027, two of the RT 017, and one of the RT 078 as well as a multi-resistant RT 012 strain. The analysis of horizontal gene transfer events revealed gene acquisition incidents that sort the strains within the time line of the spread of their RTs within Germany. We could show as well that horizontal gene transfer between the members of different RTs occurred within this multiple infection. In addition, acquisition and exchange of virulence-related features including antibiotic resistance genes were observed. Analysis of the two genomes assigned to RT 027 revealed three single nucleotide polymorphisms (SNPs) and apparently a regional genome modification within the flagellar switch that regulates the *fli* operon.

**Conclusion:**

Our findings show that (i) evolutionary events based on horizontal gene transfer occur within an ongoing CDI and contribute to the adaptation of the species by the introduction of new genes into the genomes, (ii) within a multiple infection of a single patient the exchange of genetic material was responsible for a much higher genome variation than the observed SNPs.

## Background

*Clostridioides difficile* is a Gram-positive, obligate anaerobic spore-forming bacterium, which can act as nosocomial human pathogen colonizing the intestinal tract and causing disease [1]. Symptoms of *C. difficile* infection (CDI) can range from mild diarrhea to pseudomembranous colitis or life-threatening toxic megacolon [2, 3]. *C. difficile* has been described by Miller et al. in 2011 [4] together with methicillin*-*resistant *Staphylococcus aureus* (MRSA) as the most common cause of nosocomial infections in the United States. Consequently, *C. difficile* was prioritized in the highest rank for surveillance and epidemiological research [5].

Strains of *C. difficile* are currently distinguished by PCR ribotyping (comparison of pattern of PCR products of the 16S–23S rRNA intergenic spacer region), which allows to follow epidemic infection routes [6]. Ribotype (RT) 027 was responsible for dynamic increase of CDIs in North-America and Europe, which quadrupled the number of CDI victims between 2004 and 2007 [7]. However, this epidemic outbreak had been successfully controlled with a decrease in CDI infection in 2008 [8, 9]. The infection line and the spread of the outbreak in Germany has been traced by a genome sequence-based BEAST-analysis [10]. Thus, the mutation rate of the strains challenged by the immune system of the patients was sufficient to track the transmission of the pathogen *C. difficile* from patient to patient. The dynamic adaptation of pathogens challenged by a host immune defense is the reason why host/pathogen systems have been used as model-system to investigate the speed of genome evolution [11, 12]. Genome analysis of representative strains of *C. difficile* from the PCR ribotypes 001, 017, 027 and 078 revealed the presence of distinct genomes [7]. The investigated genomes contain an extensive pan-genome shaped by horizontal gene transfer (HGT). The quantification of HGT events revealed that 11% of the whole genome is consisting of mobile elements [7, 13–15]. The described genome diversity of the species contrasts with the phenotypic similarity of isolated strains with respect to growth, virulence and pathogenicity [14]. Notably, the genes encoding the toxins TcdA and TcdB assigned to the CDI virulence [16, 17] are located on the pathogenicity locus (PaLoc) which constitutes a genomic island. This indicates that HGT is involved in the evolution of toxigenic *C. difficile* strains [18]. In *E. coli,* HGT events between different strains have been identified as an important source for new combination of virulence factors and thus for the emergence of novel pathotypes [19]. In CDI cases, multiple infections by *C. difficile* strains, which are different in morphology and virulence, have been observed [20, 21], thereby indicating the opportunity to exchange genetic material through HGT events between the strains involved. In this study, we present a comparative phenotypic and genome analysis of three morphologically different *C. difficile* strains isolated from a single patient. Two of the strains belong to RT 027 (DSM 27638 and DSM 27640) and one to RT 012 (DSM 27639).

## Methods

### Isolation of strains

A stool sample from a patient with diarrhea was cultivated anaerobically on *Clostridium difficile* (CLO) agar plates (bioMérieux, Nürtingen, Germany), which is a selective medium for *C. difficile*. After 48 h of cultivation at 37 °C, colonies characteristic for *C. difficile* were visible and were confirmed by MALDI-TOF mass spectrometry (Biotyper, Bruker Daltonics, Bremen, Germany) with score values of ≥2000. Isolated strains were deposited at the DSMZ under the accession numbers DSM 27638, DSM 27639 and DSM 27640.

### Phenotypic characterization

#### General growth conditions

*C. difficile* strains were grown at 37 °C in an anaerobic chamber (Coy Laboratory Products, Michigan USA) under an atmosphere of 5% CO_2_, 5% H_2_, 90% N_2_ or in a jar (Merck, Darmstadt, Germany) using an anaerobic gas pack (bioMérieux, Nürtingen, Germany). For liquid cultures, BHIS (brain heart infusion; BD, Heidelberg, Germany) supplemented with 0.5% yeast extract (BD, Heidelberg, Germany) and 0.03% L-cysteine (Sigma, Taufkirchen, Germany) was used. Cultivation on plates was performed using chromID™ *C. difficile* agar (CDIFF), *Clostridium difficile* agar (CLO) or Columbia agar with 5% sheep blood (COS) (bioMérieux Nürtingen, Germany). Plates were incubated for 24 to 48 h.

### Sporulation assay

Sporulation rates were determined according to Burns et al., [22]. Briefly, an overnight culture of the respective strain was diluted 1:100 with fresh BHIS and was grown until an optical density (OD_600_) of 0.2 to 0.4. This culture was again diluted 1:100 with fresh BHIS and cultivated for five days. An aliquot of the culture was heated for 25 min at 60 °C to kill the vegetative cells. Dilution series of an untreated and the heated sample with sterile saline was performed and spotted on CDIFF plates. Colony forming units (CFU) were analyzed after 24 h of incubation.

### Co-cultivation of *C. difficile* strains

An overnight culture was adjusted to an OD_600_ of 0.1 in fresh BHIS. 400 μl of the cells were incubated in one well of a 24-well plate (Greiner Bio-One, Frickenhausen, Germany). A ThinCert™ insert (pore size 0.4 μm) was placed in this well and 400 μl of the respective *C. difficile* strain was added. The insert allows the diffusion of metabolites between the two cultures but not of cells or spores. A dilution series of the cells at the time point zero was spotted on CDIFF plates to ensure that equal amounts of the respective strains were used for co-cultivation. After 24 h of incubation, the cells in the wells and inserts were resuspended thoroughly and adjusted to equal volumes. A dilution series of the cells was performed on CDIFF agar plates and incubated for 24 h. To assess whether the insert membrane is tight for *C. difficile*, controls were performed with (i) bacteria in the insert and sterile medium in the well and (ii) sterile medium in the insert and bacteria in the well. After plating aliquots on CDIFF plates, no colonies were formed after a 24 h incubation time for the medium controls, indicating that bacteria do not pass the insert membrane in relevant numbers within the 24 h incubation time.

### Mobility assay

The motility of *C. difficile* strains was tested by stab-inoculation of a fresh single colony grown on BHI-agar in 0.175–0.3% semi-solid BHI-agar. Anaerobic incubation at 37 °C, and monitoring of the developed diffusion radius around the inoculation stab for the following days, were performed. 0.3% BHI-agar plates were prepared and anaerobically incubated for 3 h before inoculation. Plates were inoculated in the center of the plate with a single fresh *C. difficile* colony and incubated under anoxic condition (5% H_2_, 5% CO_2_, 90% N_2_) at 37 °C. 1 and 2 d post inoculation, plates were removed from the anaerobic atmosphere for scanning of the plates. Motility assay on agar plates was performed incubating plates upside down and with the lid upturned to avoid problems with condensing water. Hungate tubes containing semi-solid 0.175% BHI-agar were incubated anaerobically overnight before inoculation. Agar was inoculated in the center of the hungate tube with a single fresh *C. difficile* colony using an inoculation needle in four replicates and incubated anaerobically (5% H_2_, 95% N_2_) at 37 °C. Diffusion radius around the inoculation stab was monitored taking pictures 1, 2 and 3 d post inoculation.

### Transmission electron microscopy

For visualization of *C. difficile* cells via Transmission Electron Microscopy (TEM) cells were negatively stained using 1% (*w*/*v*) uranyl acetate. *C. difficile* cultures were inoculated and grown to exponential or stationary phase. Cultures were either used directly for sample preparation, or were previously washed to get rid of media ingredients. Therefore 2 ml liquid culture were centrifuged at 4000 rpm for 5 min, washed with 1 ml 50 mM Tris and centrifuged again. Subsequently, the pellet was resolved in 200 μl Tris. For sample preparation, an EM S160–3 cupper grid was incubated on a droplet of liquid *C. difficile* culture, or washed cells, for 1 min to allow absorption of cells to the grid’s carbon film. The grid was carefully semi-dried with a filter, preventing crystallization of media ingredients on the carbon film. The grid was washed in a droplet of deionized H_2_O, filter-dried and negatively stained on a droplet of 1% (w/v) uranyl acetate solution for 15 s. Afterwards the grid was completely dried with a filter and analyzed using a Jeol JEM-1011 TEM.

### Antibiotic resistance susceptibility tests

Susceptibility to metronidazole, erythromycin, vancomycin, rifampicin and moxifloxacin was performed using Etest® strips (bioMérieux, Nürtingen, Germany). Grown cells (according the described general growth conditions above) were adjusted with 0.9% saline to a McFarland standard 1 and swabbed onto Mueller-Hinton agar supplemented with 5% horse blood and 20 mg/l β-NAD^+^ (bioMérieux, Nürtingen, Germany). Plates were incubated anaerobically at 37 °C and MIC breakpoints were read after 48 h. Control strains (Table 1) were included to verify the reproducibility of the test.

**Table 1:** Antibiotic resistance patterns of the three *C. difficile* strains

Isolate	Rifampicin	^b^Vancomycin	^b^Metronidazole	^c^Moxifloxacin	Erythromycin
DSM 27638	^a^0.004	S (0.5)	S (0.38)	R (>32)	R (>256)
DSM 27639	S (0.003)	S (0.75)	S (0.25)	S (2)	R (>256)
DSM 27640	^a^0.004	S (0.38)	S (0.25)	R (>32)	R (>256)
DSM 27543	S (0.003)	S (0.5)	S (0.25)	S (2)	R (>256)
DSM 27147	^a^0.004	S (0.5)	S (0.38)	R (>32)	R (>256)

May be tested for epidemiological purposes only (ECOFF 4 mg/L (EUCAST Clinical Breakpoint Table v. 7.1.). DSM 27543 (630) and DSM 27147 (R20291) served as controls

MIC given in mg/mL, *S* sensitive, *R* resistant

^a^Not used clinically. May be tested for epidemiological purposes only (ECOFF 0.004 mg/L). Vancomycin: *R* > 2 mg/L. Metronidazole: R > 2

^b^The breakpoints are based on epidemiological cut-off values, which distinguish wild-type isolates from those with reduced susceptibility

^c^Not used clinically

### DNA extraction, genome sequencing, de novo genome assembly and genome annotation

For genome sequencing the strains were cultivated anaerobically in Wilkins-Chalgren Anaerobe Broth (Oxoid, Basingstore, United Kingdom) at 37 °C. Genomic DNA was extracted as described previously [23, 24].

Genome sequencing of the *C. difficile* strains was carried out on the PacBio *RSII* (Pacific Biosciences, Menlo Park, CA) using P5 chemistry. Genome assembly was performed with the RS_HGAP_Assembly.3 protocol included in SMRT Portal version 2.3.0. The chromosomal contigs generated were trimmed, circularized, and adjusted to *dnaA* as the first gene.

In parallel, genome sequencing of the *C. difficile* strains was carried out on a Genome Analyzer GAIIx (Illumina, San Francisco, CA) in a 112 bp paired-end single-indexed run Quality improvement of the final consensus sequence was performed with the Burrows-Wheeler Aligner (BWA) using bwa aln and bwa sampe [25] mapping the Illumina reads onto the obtained chromosomal contigs from the PacBio sequencing. A final quality score of QV60 was attained. Automated genome annotation was carried out using Prokka [26]. Subsequentially, selenocysteine proteins were annotated manually. Complete genome sequences have been submitted to GenBank under the accession numbers CP011846.1 (DSM 27638), CP011847.1 (DSM 27639) and CP011848.1 (DSM 27640). No extrachromosomal genetic elements were observed within this sequencing study.

### Comparative genomics

The genomes of strains DSM 27638, DSM 27639 and DSM 27640 were compared with finished closed references genomes selected as members of the corresponding RTs, Comparative genomics included three strains belonging to the most virulent RT 027 (non-epidemic strain CD196, the epidemic and highly virulent strain R20291 [14], and the bovine isolate 2,007,855), three other strains belong to recently emerging RTs 017 (CF5 and M68) and RT 078 (M120). The RT 012 is represented by strain 630 [23, 27]. All analyzed strains are listed in Table 2. Orthologous proteins were determined with ProteinOrtho [28] applying default parameters. Circular visualizations and comparisons of shared nucleotide regions of the genomes have been produced with BRIG [29] and linear visualizations with MAUVE [30]. Identification of genomic islands has been done with Island viewer 3 [31]. All identified regions have been manually curated using UniProtKB/Swiss-Prot and TrEMBL database (www.uniprot.org). In detail, comparison of related genome regions have been done with ACT and Artemis [32]. Phylogenomics based on whole genome alignments has been performed by using Phylomark [33]. Synteny analysis and SNP prediction have been performed with nucmer from the mummer program suite [34].

**Table 2:** Genome sequences used in this study

Strain	Ribotype	Toxino-type	Genome size	Isolation (year/country)	Accession number	Reference
DSM 27638^a^	027	III	4,229,698	2015/Germany	CP011846.1	This study
DSM 27639^a^	012	0	4,263,997	2015/Germany	CP011847.1	This study
DSM 27640^a^	027	III	4,229,629	2015/Germany	CP011848.1	This study
630^a^	012	0	4,274,782	1982/Switzerland	CP010905.2	[27]
CF5^b^	017	VIII	4,159,517	1995/Belgium	FN665652.1	[14]
M68^b^	017	VIII	4,308,325	2006/Ireland	NC_017175.1	[14]
CD196^c^	027	III	4,110,554	1985/France	NC_013315.1	[49]
R20291^d^	027	III	4,191,339	2006/UK	FN545816.1	[14]
2007855^d^	027	III	4,179,867	2007/US	FN665654.1	[14]
M120^b^	078	V	4,047,729	2007/UK	NC_017174.1	[51]

^a^PacBio/Illumina hybrid assembly

^b^454/Illumina hybrid assembly

^c^454/Sanger sequencing

^d^454 sequencing

## Results and discussion

### Phenotypic characterization

**Fig. 1:**
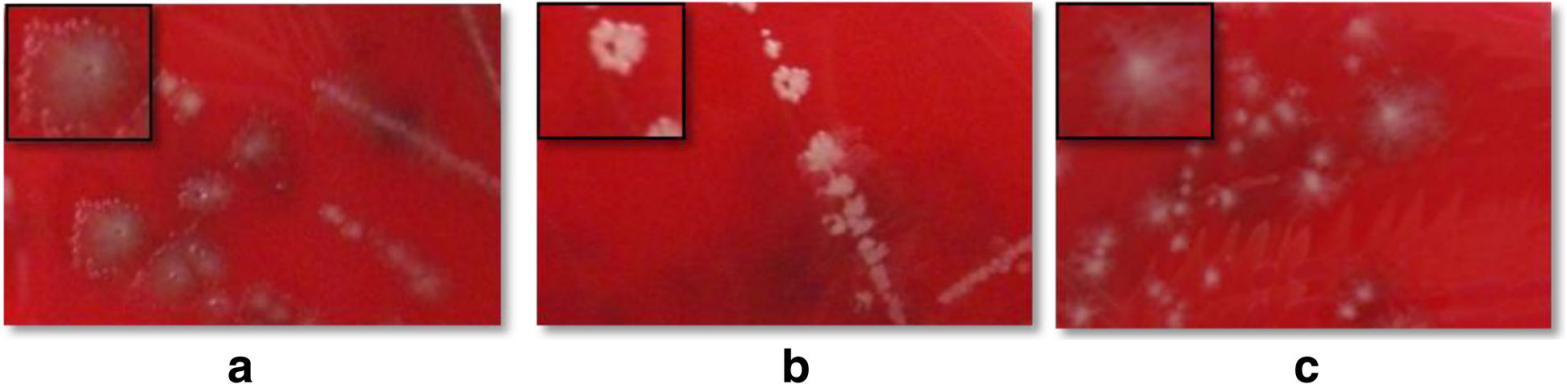
Colony morphology of *C. difficile* strains*.* Strains were grown anaerobically (5% H_2_, 95% N_2_) on commercial *Clostridium difficile* agar (CLO) for 2 days at 37 °C. DSM 27638 (**a**) and DSM 27640 (**c**) show irregular colony shape with shiny surfaces and white color. Colonies from DSM 27640 (**c**) are more opaque than colonies from DSM 27638 (**a**). Colony shape of DSM 27639 (**b**) looks nearly filamentous, colony surface is smooth and the color is cloudy and whitish

Three *C. difficile* strains (DSM 27638, DSM 27639 and DSM 27640) exhibiting different phenotypes on solid medium have been isolated from one stool sample of a single patient suffering from CDI. The isolate DSM 27639 formed colony types that were white and smooth with clearly defined edges (Fig. 1). The other two isolates DSM 27638 and DSM 27640 had a rougher surface and seemed to spread on the agar plate (Fig. 1). Both isolates mainly differed in color; isolate DSM 27638 was grayish compared to isolate DSM 27640 which appeared rather gray beige. This initial observation indicated that the patient was infected with more than only one *C. difficile* strain. Since it has been reported that multiple infection with pathogens like *C. difficile* occur [20, 21], we aimed to more precisely characterize the respective phenotypic and genotypic differences that occurred during this multiple infection.

Toxinotyping has been used for distinguishing *C. difficile* strains. In this regard, toxin A and B genes located on the PaLoc are considered to be the major virulence factors of *C. difficile*. Sequencing of the complete genomes showed that the PaLocs from all three isolates were intact. As expected, the toxin loci of DSM 27638 and DSM 27640 grouped to toxinotype III, which is observed for strains belonging to RT 027 [35]. The toxin locus of isolate DSM 27639 belonged to toxinotype 0, which correlated with strains grouping into RT 012 [35].

Both RT 027 isolates DSM 27638 and DSM 27640 exhibited the typical antimicrobial susceptibility pattern of this RT, including high resistance against erythromycin and moxifloxacin (Table 1). In contrast, the RT 012 isolate DSM 27639 was susceptible to moxifloxacin. All isolates were susceptible to vancomycin and metronidazole that are antibiotics commonly used in CDI therapy [36].

It has been reported that multiple *C. difficile* strains can co-exist in an in-vitro human gut model although exhibiting different growth rates [37], we investigated the growth behavior of the three isolates under pure culture conditions as well as under co-cultivation conditions. However, the strains showed an identical growth behavior in BHIS under standard conditions (Additional file 1: Figure S1) In addition, all strains had the same maximal sporulation ability shown by comparable amounts of germinated spores on plates after incubation under harsh nutrient starvation (Additional file 2: Figure S2). Co-cultivation of the isolates showed that none of them had either a negative or a positive effect on the growth of any of the other isolates or the growth of reference strain 630 (data not shown). The absence of intra-species competition most likely has supported their co-existence in the patient. However, those in vitro results obviously cannot reflect the complex situation in the human host where nutrients are limiting and the different isolates are challenged by the host immune system and complex gut microbial community.

**Fig. 2:**
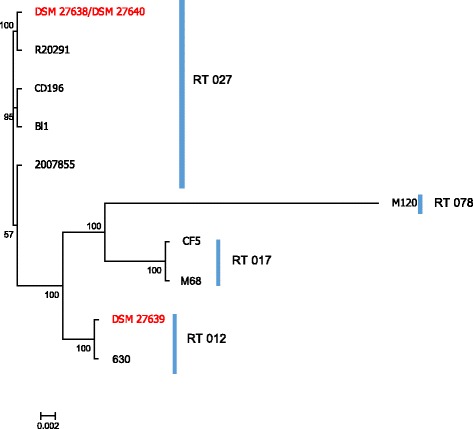
Whole genome alignment based phylogenomic tree. Strain DSM 27639 clusters with reference strain 630 and strains DSM 27638/40 with all RT 027 strains. The tree has been calculated using Phylomark with default parameters. The clinical isolates are marked in red, due to the extreme low editing distance the node of DSM 27638 and DSM 27640 has been collapsed

The clinical isolates DSM 27638, DSM 27639 and DSM 27640 exhibit representative phenotypic features of the toxinotype they belong to which is confirmed by the phylogenetic clustering based on whole genome sequence comparison (Fig. 2). However, phenotypic analysis could not explain the presence of two RTs in one patient.

### General genome comparison

To determine the genomic features correlating with the observed colony phenotypes we performed complete genome sequencing of all three isolates. Their genomes were compared with seven publicly available closed *C. difficile* genomes including reference genomes of four different PCR ribotypes. A whole genome alignment using Phylomark [33] was used to assign strains DSM 27638 and DSM 27640 to RT 027 and the isolate DSM 27639 to RT 012 (Fig. 2). The genome alignment of these isolates and reference strains using MAUVE showed that all *C. difficile* strains share a complete syntenic chromosome interrupted by mobile elements, as it has been observed in other virulent clostridia [38] (Additional file 3: Figure S3).

**Fig. 3:**
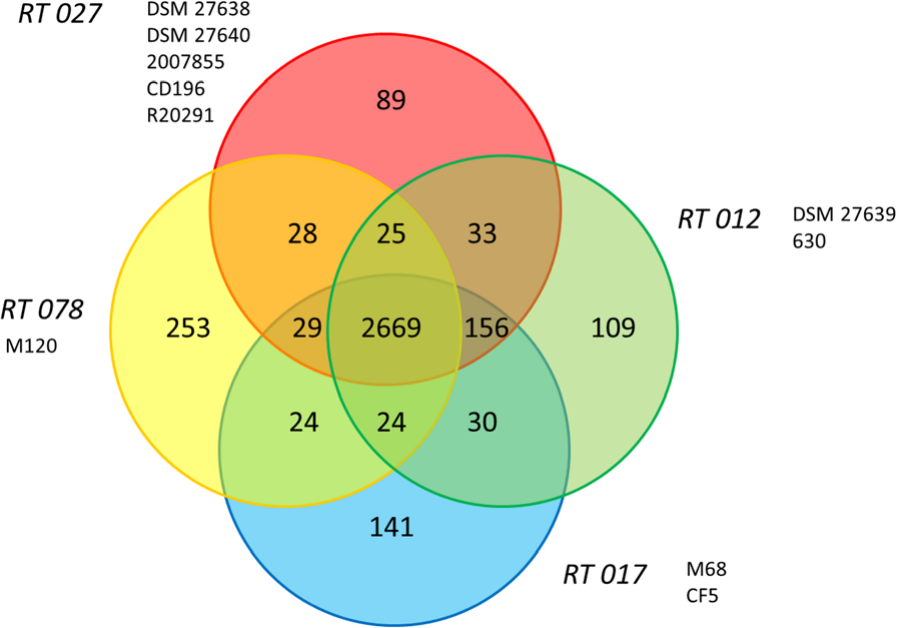
Core/Pan-genome calculation of the RT 012, RT 017, RT 027, and RT 078. The four ribotypes share a core genome of 2669 genes. The genomes used to generate the protein datasets are indicated. Orthologous proteins have been identified with ProteinOrtho software (Lechner et al., [28])

To determine the core genome orthologous coding sequences (CDS) between all *C. difficile* strains were identified. Thus, we identified a core genome of 2669 CDS shared by all strains (Fig. 3). Consistent with the antibiotic dependent pathogenicity of CDI the core genomes comprises a number of genes assigned to antimicrobial resistances (Additional file 4: Table S1), including the beta-lactamase-inducing penicillin-binding protein BlaR; the quaternary ammonium compound-resistance protein SugE, and the vancomycin/teicoplanin-resistance proteins VanG, VanV and VanW. Resistance of *C. difficile* against the fluoroquinolone moxifloxacin is characteristic for most RT 027 strains and provides them with a selective advantage in comparison to other ribotypes when this antibiotic is used. The historical RT 027 strain CD196 in contrast to recently isolated RT 027 strains is moxifloxacin susceptible [39]. It was already shown that a single point mutation in the DNA gyrase subunit A-encoding gene *gyrA* of *C. difficile* leads to fluoroquinolone resistance [40]. In contrast to *C. difficile* reference strain 630 and isolate DSM 27639, the RT 027 strain 2,007,855 and the isolates DSM 27638 and DSM 27640 are resistant to fluoroquinolones. Sequence analysis of the GyrA protein confirmed that all moxifloxacin-resistant *C. difficile* strains contain a single transition mutation resulting in the amino acid substitution Thr-82-Ile (Additional file 5: Figure S4) [41].

**Fig. 4:**
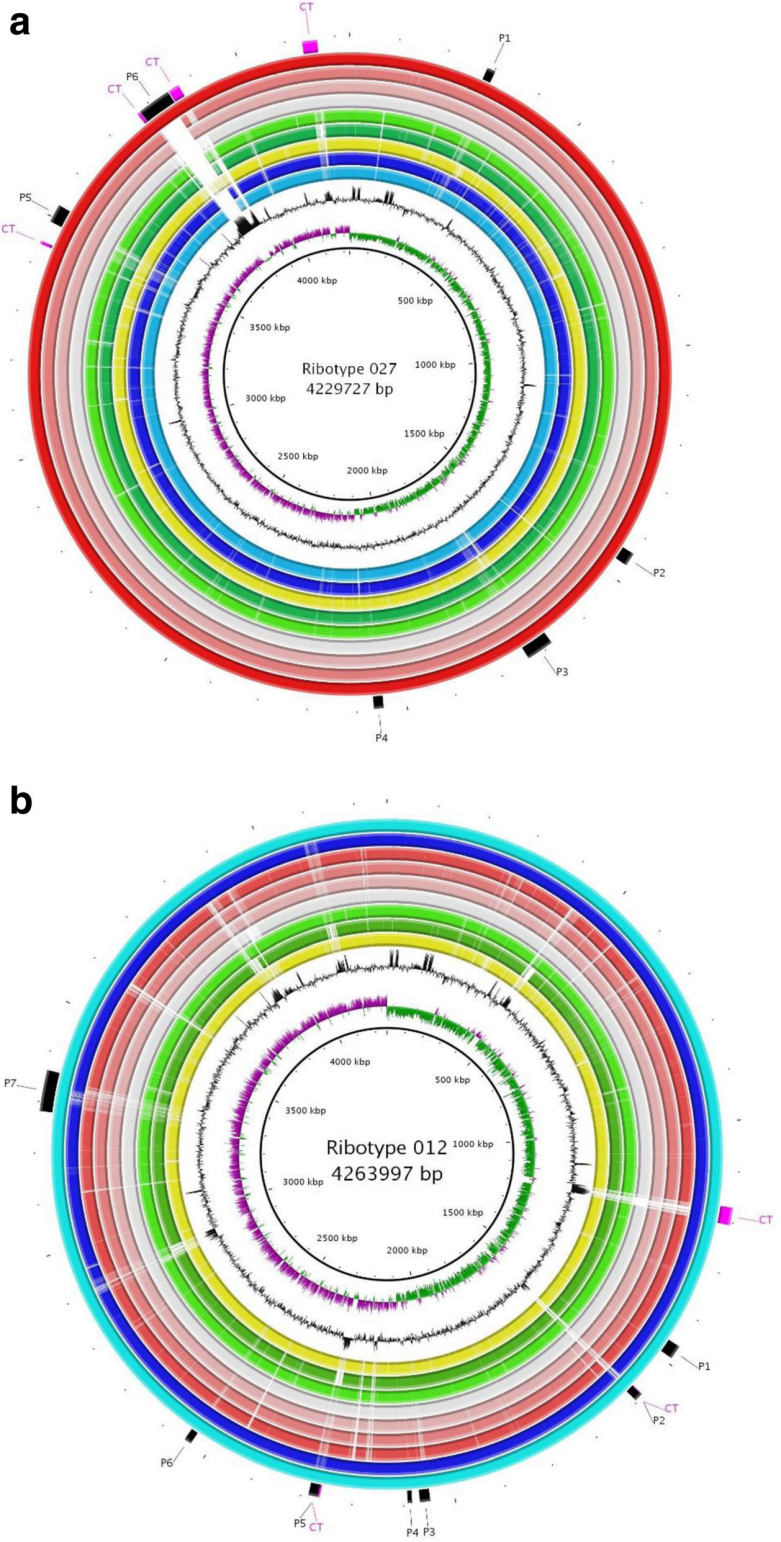
Circular representations of *C. difficile* chromosomes generated with BRIG software. From the outside: circle 1 shows identified mobile elements (black – prophages P1–7, pink – conjugative transposons CT). Circle 2 shows reference genome **a** strain DSM 27638 / DSM 27640, **b** strain DSM 27639). The most inner circle represents scale (in kb), second inner – GC skew for reference genome, third – GC content graph. Inner rings represent analyzed genome sequences of red colors ribotype 027 (the most dark red: DSM 27638 then 2,007,855, R20291, and light red 196); blue colors represent 012 ribotype (dark blue: 630, light blue: DSM 27639); green colors represent ribotype 017 (dark green: CF5, light green: M68) and yellow color represent strain M120 belonging to RT 078

In addition, strain-specific and ribotype-specific CDS were identified. The locations of regions of genetic difference between the strains are highlighted in the concentric circular chromosome representations of the analyzed ten genomes (Fig. 4). Strain specific genes are often found to be encoded in regions that have been identified as prophages or conjugative transposons (Table 3).

**Table 3:** Mobile elements of strains DSM 27638 and DSM 27639^a^

Start	Stop	Length [bp]	ORFs	Mobile element and gene content
DSM 27638				
284,586	301,831	17,244	16	PHAGE (P1) (not found in 012 and 017 ribotypes)
398,255	402,824	4569	7	ABC transporter, two-component system, transposase
501,871	511,046	9229	10	transposase, hydrolases, transcriptional regulator, oligo-1,6-glucosidase, PTS system transporter subunit IIABC
518,083	528,817	10,734	10	lantibiotic resistance, two two-component systems, ABC transporter
662,517	674,897	12,380	11	ABC transporter, two-component system, transcriptional regulator
934,705	938,946	4241	5	lantibiotic resistance two-component system, two-component system
1,438,093	1,465,397	27,304	30	PHAGE (P2)
1,680,933	1,736,907	55,974	69	PHAGE (P3) (ribotype 027 specific)
2,046,513	2,066,289	19,776	6	PHAGE (P4) (ribotype 027 specific)
3,441,029	3,447,002	5973	6	conjugative transposon
3,478,606	3,491,098	12,492	8	integrase, chromosome segregation ATPase
3,489,961	3,527,152	37,191	26	PHAGE (P5) (ribotype 027 specific)
3,769,213	3,775,340	6127	7	conjugative transposon
3,775,480	3,844,734	69,254	54	PHAGE (P6) (DSM 27638 specific)
3,846,878	3,869,593	22,715	22	conjugative transposon
4,100,257	4,105,353	5096	5	ABC transporter, two-component system
4,134,037	4,164,119	30,082	34	conjugative transposon, multidrug resistance protein (ribotype 027 specific)
DSM 27639				
1,170,734	1,205,103	34,369	33	conjugative transposon, lantibiotic ABC transporter (DSM 27639 specific)
1,461,445	1,488,797	27,352	30	PHAGE (P1)
1,578,539	1,595,263	16,724	17	conjugative transposon (DSM 27639 specific)
1,580,053	1,595,960	15,907	9	PHAGE (P2) (DSM 27639 specific)
2,047,626	2,068,044	20,418	7	PHAGE (P3) (ribotype 012 specific)
2,083,042	2,091,718	8676	9	PHAGE (P4)
2,263,687	2,285,988	22,301	26	conjugative transposon (DSM 27639 specific)
2,267,275	2,285,716	18,441	11	PHAGE (P5) (DSM 27639 specific)
2,536,302	2,550,493	14,191	23	PHAGE (P6) (ribotype 012 specific)
3,282,029	3,362,995	80,966	84	PHAGE (P7) (DSM 27639 specific)

^a^DSM 27,640 is not included because it is identical to DSM 27638 in all elements. All mobile elements have been predicted by IslandViewer 3, PHASTER and have been manually curated

The overall similarity strongly underlines that RT 027 strains are closely related. Sequence data show that six genetic regions (two transposons and four prophages P1, P3, P4, and P5) are unique to the RT 027. The biggest difference observed in the genome of DSM 27638 is the presence of a predicted prophage integrated at 3.78 to 3.84 Mbp flanked by conjugative transposons (Fig. 4, Table 3). Interestingly, there is a conjugative transposon present at position 3.84 to 3.87 Mbp shared by the strains DSM 27638 and DSM 27640 as well as strain R20291 but not by the RT 027 reference strain CD196. This indicates that the four strains might share a common history starting at a RT 027 ancestor that did not contain the conjugative transposon. The ancestor of strains DSM 27638, DSM 27640 and R20291 might have acquired the transposon locus, which in the ancestor of strains DSM 27638 and DSM 27640 was the integration locus of a prophage.

Sequence comparison of two RT 012 genomes (630 (DSM 27543) and DSM 27639) revealed a high degree of synteny except for those regions that encode mobile elements (Additional file 3: Figure S3). A circular BLAST based comparison of the DSM 27639 genome focused on the RT 012 genomes revealed that the difference between both genomes correlates directly to predicted prophages and conjugative transposons (Fig. 4 b). Two prophages (P3 and P5) and five transposons are shared exclusively by the genomes of the strains DSM 27639 and 630 and might therefore be acquired by an RT 012 ancestor. The remaining two prophages (P1 and P4) are specific for strain DSM 27639. One prophage region is shared by all nine analyzed strains.

Comparative genomics revealed that the biggest difference of the strains isolated from the same patient to the reference genomes is the acquisition or loss of prophages and conjugative transposons, which fits to the observations of Hargreaves et al. and Mullany et al. [18, 42]. Thus we conclude that the strain-specific genes are a result of acquisition of mobile elements, which indicates the importance of these elements for the emergence of virulence. In contrast to the prophage regions, all conjugative transposons correlate with GC-content variations compared to the average GC-content (see Fig. 4). This indicates that the acquisition of the transposons are a more recent event and thus the forces of amelioration of newly acquired genome regions (described in [43] and references therein) to the host genome have had less time to operate.

### In *infectio* genome dynamics of DSM 27638 and DSM 27640

**Fig. 5:**
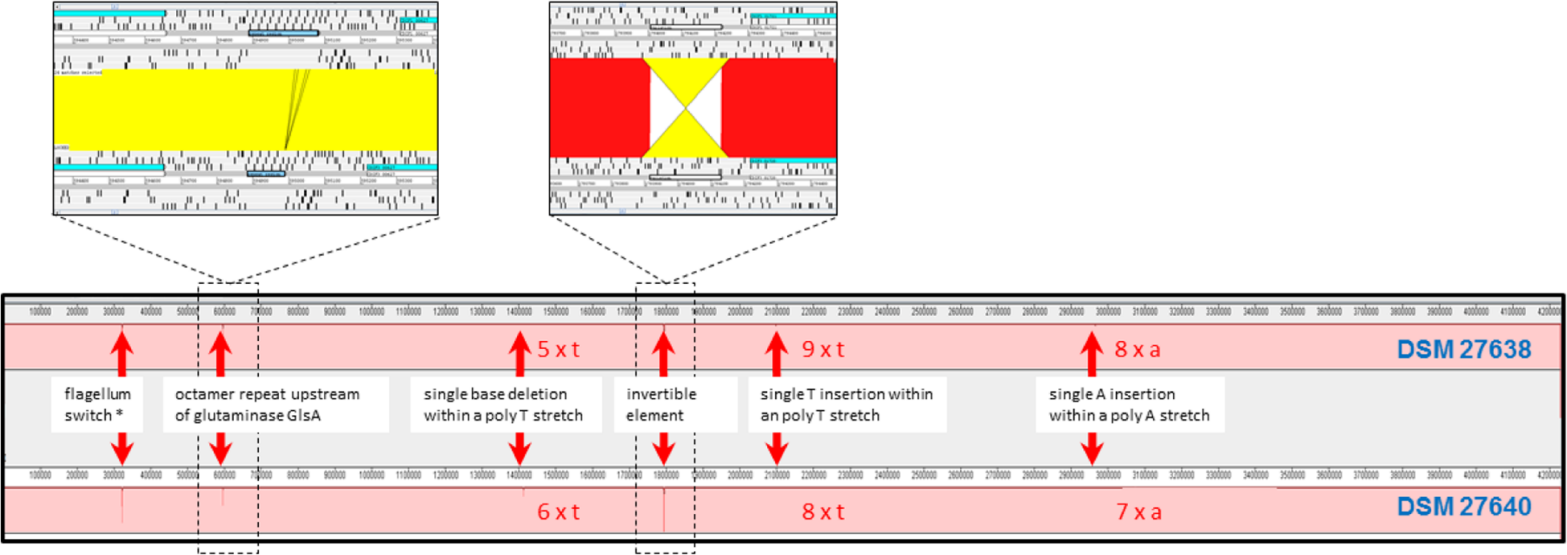
Sequence differences of *C. difficile* isolates DSM 27638 and DSM 27640. Three regions out of six are point mutations. The flagellum switch region is described in detail in Fig. 7. The octamer repeat region and an invertable element are presented in detail

The genome sequences of the two RT 027 isolates DSM 27638 and DSM 27640 are almost identical (Additional file 6: Figure S5), which suggest a clonal history of them within the patient [14, 44]. The isolates encode the same number of predicted proteins and differ by only 69 bp in size (Table 2, Fig. 5). A whole genome BLAST comparison of the genomes revealed the differences within six genome regions five of them being found within intergenic regions (Table 4). Two loci represent imperfect inverted repeats upstream of operons, a motif which has been found in *Salmonella enterica* as regulatory element where the inverted repeat regulates the downstream operon upon inversion [45]. Johnson described the regulation as a reversible flip/flop mechanism. Three inverted loci flanked by inverted repeats have been described as well as a difference between the originally published of the *C. difficile* genome 630Δ*erm* and a high quality re-sequenced version in Dannheim at el. [27] indicating a reversible nature of these genomic elements. However, a comparison of the reverse complement sequence from isolate DSM 27638 with the sequence of DSM 27640 revealed that the regions differ in six base positions between the two strains. In contrast to the 630Δ*erm* strains the locus differs between strains DSM 27638 and DSM 27640 not only in a reversible inversion. The locus is located upstream of the first CDS of the late flagellum genes and has been investigated in detail by Anjuwon Foster et al. [46] who named the regulatory element as flagellum switch. The sequence of strain DSM 27638 is identical to the 154 bp sequence described for RT 027 in contrast to the DSM 27640 version that contains additionally 4 bp. In contrast, the second inverted repeat, which is located upstream of a diguanylate cyclase, exhibits no difference compared to the reverse complement sequence of the corresponding locus of strain DSM 27640. This observation and the possibility that this kind of inverted repeat may be reversibly inverted [45, 47] challenges the hypothesis that the two genome regions really can be considered as different. Furthermore, the operons located adjacent to the inverted repeat encode transporters where a possible contribution to a macroscopic visible strain difference is at least not obvious. The most prominent sequence difference between the two isolates is generated by the insertion of eight instances of an octamer repeat-unit in isolate DSM 27638 at position 594,943 to 595,006. This 64 bp insertion is responsible for almost the complete size difference of 69 bp of the two genomes (Table 2). As a result DSM 27638 contains 21 repeat-units and DSM 27640 13 repeat-units at the corresponding locus. The repetitive region is located within the intergenic region of a locus that encodes several genes assigned to spore surface components. However, although it is possible that a modification of the regulation of spore surface components might result in the observed phenotypic differences a comparative investigation of the sporulation behavior and the viability of the spores revealed no significant differences between the three isolates (Additional file 2: Figure S2). Since the two isolates are - in contrast to the well investigated reference strain 630 - not yet genetically accessibility, a systematic investigation of the multiple repeat region and its putative contribution to the observed differences of growth phenotypes is not possible to date. The remaining genome sequence differences are three single base insertion, two of them located in intergenic regions and only one impacts an encoding signal peptidase.

**Table 4:** Sequence differences of *C. difficile* isolates DSM 27638 and DSM 27640

DSM 27638	DSM 27640	Description
321,766 to 321,922	321,766 to 321,918	six base variations within the flagellum switch, an invertible element downstream of a c-di-GMP riboswitch within the 5’UTR of the *flgB*-gene
594,884 to 595,051	594,880 to 594,983	octamer repeat upstream of glutaminase GlsA, DSM 27638 contains eight additional instances
. Gap at 1,411,589	T at 1,411,522	single base deletion within a poly T stretch generates a disfunctional signal peptidase Sip3 gene in DSM 27638
1,793,982 to 1,794,196	1,793,915 to 1,794,129	invertible element upstream of signaling protein (CDIF27640_01720 resp. CDIF27638_01720). The reverse complement of the DSM 27638 sequence is identical to the DSM 27640 sequence
T at 2,096,993	. Gap 2,096,925	single T insertion within an poly T stretch, upstream of stage V sporulation protein S SpoVS
A at 2,964,477	. Gap 2,964,408	single A insertion within a poly A stretch, upstream of a purine riboswitch regulated permease (CDIF27638_02797 resp. CDIF27640_02796)

### The *fli* locus

**Fig. 6:**
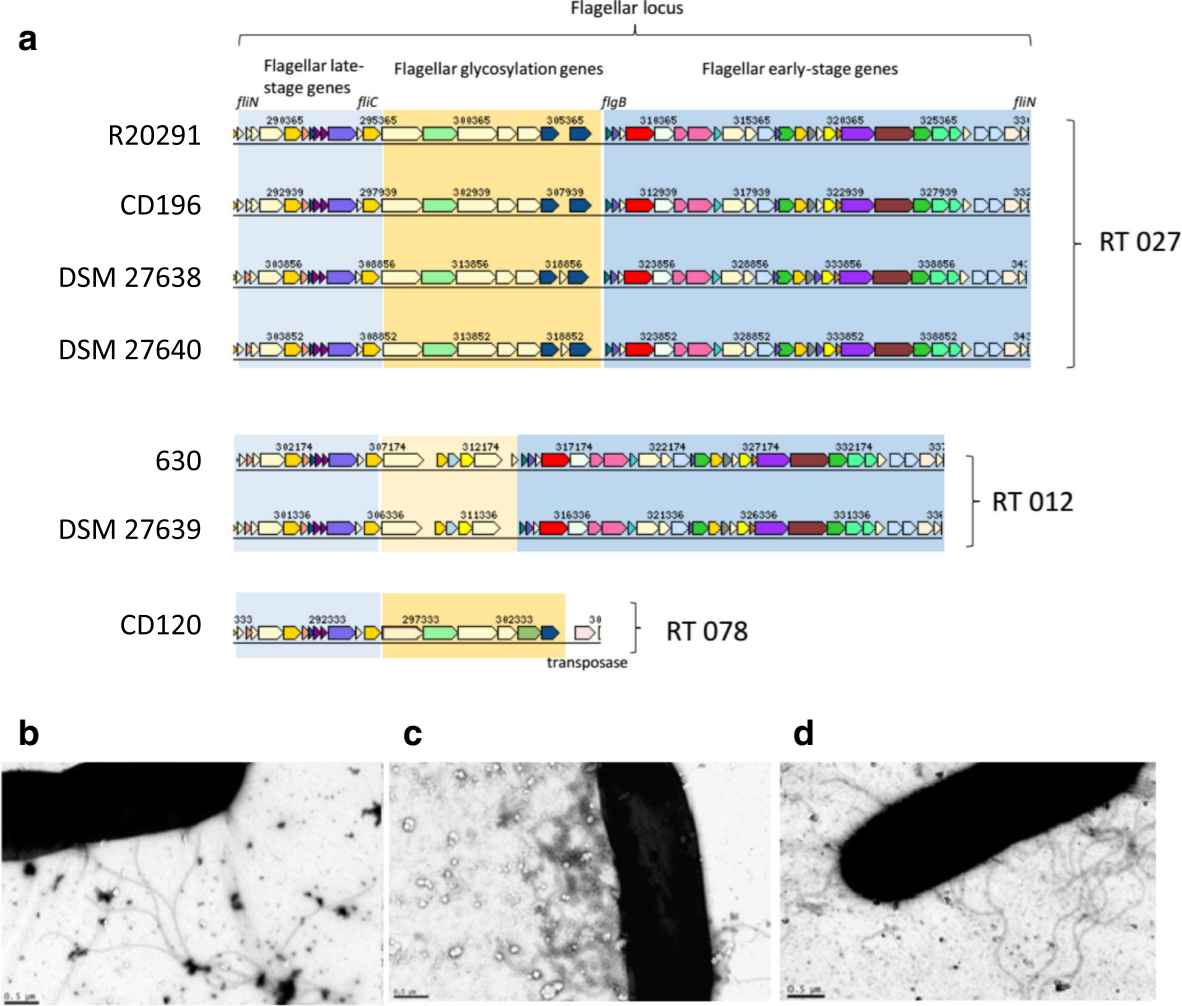
Flagella in *C. difficile* strains. **a** Arrangement of genes in the flagellar locus. **b**-**d** Transmission electron microscopy (TEM) of vegetative cells*.* The rod-shaped cells of all analyzed *C. difficile* strains appeared peritrichous flagellated indicating that the flagellar locus is functional. **b** DSM 27638; **c** DSM 27639*;* **d** DSM 27640

**Fig. 7:**
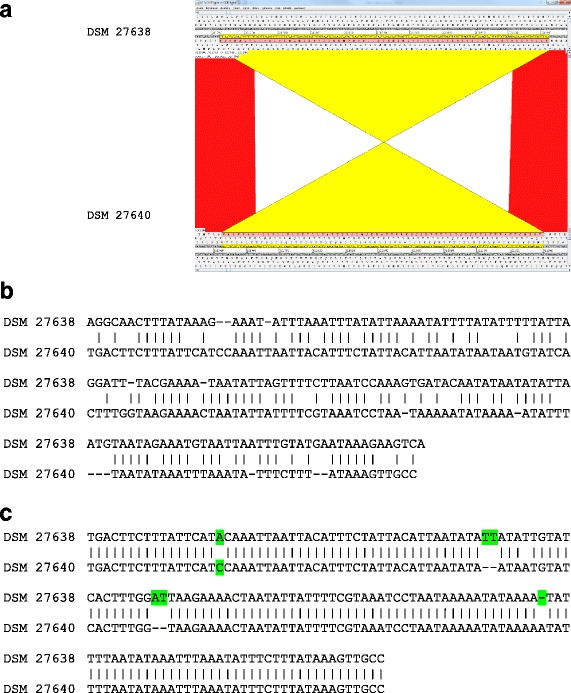
Comparison of upstream region of *flgB* in strains DSM 27638 and DSM 27640. **a** Pairwise comparison of the *C. difficile* DSM 27638 and DSM 27640 region of inversion displayed using the Artemis Comparison Tool (ACT; http://www.sanger.ac.uk/science/tools/artemis-comparison-tool-act). The red and yellow bars indicate regions of similarity with red bars indicating corresponding regions that are oriented similarly and yellow bars indicating regions oriented in opposite directions; **b** Alignment of region from DSM 27638 (321766–321,922) and DSM 27640 (321766–321,918); **c** Alignment of complement/reverse version from DSM 27640 with DSM 27638 original sequence

The *fli* locus encodes the flagellum of *C. difficile*, which results in motile peritrichous *C. difficile* cells [48]. A genome analysis of the *fli* loci of the three isolates and comparisons with RT 027 and RT 012 reference strains as well as with a non-motile RT 078 control strain confirmed that all structural genes necessary to encode a functional flagellum are present in all three isolates, which could be confirmed via TEM (Fig. 6). Note that that all amino acid sequences of the *fli*-gene products of strains DSM 27638 and DSM 27640 are identical. Since the intergenic regulatory region upstream of the early stage *fli* genes is one of only three regional sequence differences identified in the complete genome sequences of DSM 27638 and 27,640 we performed additional phenotypic investigations to verify the expression of a functional flagellum (Fig. 7). In contrast to the non-motile RT 078 control strain, both RT 027 isolates as well as the RT 012 isolate DSM 27639 and the respective control isolates R20291 (RT 027) and 630 (DSM 27543, RT 012) exhibited a spreading diffuse growth indicative for active motility (Additional file 7: Figure S6). Thus the impact of the described genome difference on the initially observed growth phenotype (Fig. 1) remains unsolved. However, it has been reported that the flagellum can have an impact on the adherence to intestinal mucosa and might eventually also influence growth on solid surfaces such as agar plates [46, 49, 50]. Thus it is tempting to speculate that the sequence difference within the *fli* locus contributes to the observed growth phenotype of the three patient isolates.

## Conclusion

The analysis of three phenotypic diverse *C. difficile* isolates that were isolated simultaneously from a stool sample of a diarrheic patient confirmed that multiple and isochronal infections with different RTs occur. The phenotypic and genetic characterization could not give an answer which strain (if that is a case) caused the CDI since all three isolates harbor apparently an intact complete PaLoc encoding the *tcdA* and *tcdB* toxin genes and there are no obvious phenotypic advantages showing that one isolate distinctly differs from the others. However, our sequence-based analysis gave insights into genome evolution in micro- and macroscale, as well as in *in infectio* adaptation. The genome history of the three analyzed isolates has been tracked by a comparative genome analysis. The acquisition/loss of prophage and conjugative transposons is most impressive. The observation that strains of different RTs within single infection have exchanged genetic material in form of mobile genetic elements indicates that genome variation might be as well an effect of a community-based maintenance of a common pan-genome which would be separate genomic adaptations from evolutionary events.

Apparently, inversion events of intergenic regions correlate to phenotypic variation. An in-depth analysis of two isolates from RT 027 indicate an *in infection* strain adaptation. Thus genome modification events which lead to phenotypic diversification and in long-term to the evolution of new strains can be observed in a single infection event.

## Additional files

Additional file 1: Figure S1. Growth curves in BHIS of DSM 27638, DSM 27639 and DSM 27640. The isolates were grown in brain heart infusion medium containing 0.5% (*w*/*v*) yeast extract und 0.03% (w/v) L-cysteine. All isolates have the same growth rate under laboratory conditions as shown as the means of three replicates with standard deviation. (DOCX 55 kb)

Additional file 2: Figure S2. Sporulation assay of DSM 27638, DSM 27639 and DSM 27640 on ChromID plates after 5 days incubation on BHIS. All three isolates show a comparable count of spores that germinated on the plate. (DOCX 275 kb)

Additional file 3: Figure S3. Mauve whole genome alignments of ten linearized *C. difficile* strains from four different PCR ribotypes. In this alignment process locally collinear blocks (LCBs) were generated. Boxes with identical colors represent LCB, indicating homologous DNA regions shared between the chromosomes without sequence rearrangement. Lines collate aligned segments between genomes. The vertical bars denote the conservation level, and upward and downward orientations relative to the genome line indicates collinear and inverted regions, respectively. The only non syntenic region of the genomes is indicated by the red box. Sequences outside colored blocks do not have homologues in the other genome. (DOCX 419 kb)

Additional file 4: Table S1. Genes assigned to resistance. (DOCX 16 kb)

Additional file 5: Figure S4. DNA gyrase subunit A alignment. The resistance phenotype is encoded by the amino acid substitution at the position 82 of GyrA. (DOCX 33 kb)

Additional file 6: Figure S5. Genome comparison of *C. difficile* isolates DSM 27638 and DSM 27640. The genome share a complete highly conserved chromosome reflected by the main diagonal. The repeats are depicted by the blue dots evenly distributed within the graph. The comparison has been calculated with Mummer 3 using default parameters. (DOCX 603 kb)

Additional file 7: Figure S6. Motility assay of *C. difficile* isolates. RT 012 strains (630; DSM 27639), RT 027 strains (R20291; DSM 27640; DSM 27638) and non-motile RT 078 (DSM 29747) as a reference were analyzed on 0.3% BHI-agar. Following 1 to 2 days of post inoculation, the diffusion radius was monitored in semi-solid hungate tubes (A) and on semi-solid agar plates (B), respectively. Note, all strains produced gas. (DOCX 11038 kb)

## Abbreviations

AAD: Antibiotic associated diarrhea
BEAST: Bayesian evolutionary analysis by sampling trees
BHI: Brain heart infusion
BHIS: Brain heart infusion supplemented
BWA: Burrows-Wheeler Aligner
C: Clostridioides
CDI: *C. difficile* infection
CDIFF: *C. difficile* agar
CDS: Coding sequences
CFU: Colony forming units
CLO: *Clostridium difficile* agar
COS: Columbia agar with 5% sheep blood
HGT: Horizontal gene transfer
MRSA: Methicillin*-*resistant *Staphylococcus aureus*
PaLoc: Pathogenicity locus
RT: Ribotype
TEM: Transmission Electron Microscopy
