# Supplementary figures and images for: Comparative genome and phenotypic analysis of three Clostridioides difficile strains isolated from a single patient provide insight into multiple infection of C. difficile
Source: BMC Genomics. 2018 Jan 2;19:1. doi: 10.1186/s12864-017-4368-0 (PMC5749029; doi:10.1186/s12864-017-4368-0)

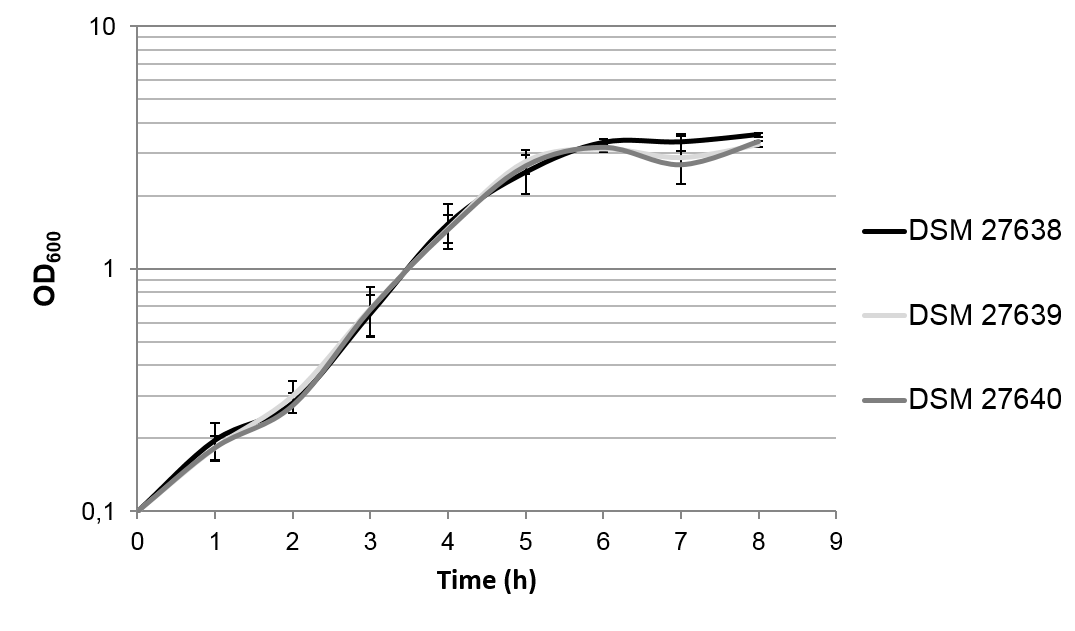

Supplement: Supplementary file 1 — Growth curves in BHIS of DSM 27638, DSM 27639 and DSM 27640. The isolates were grown in brain heart infusion medium containing 0.5% (w/v) yeast extract und 0.03% (w/v) L-cysteine. All isolates have the same growth rate under laboratory conditions as shown as the means of three replicates with standard deviation. (DOCX 55 kb) [file 12864_2017_4368_MOESM1_ESM.docx]

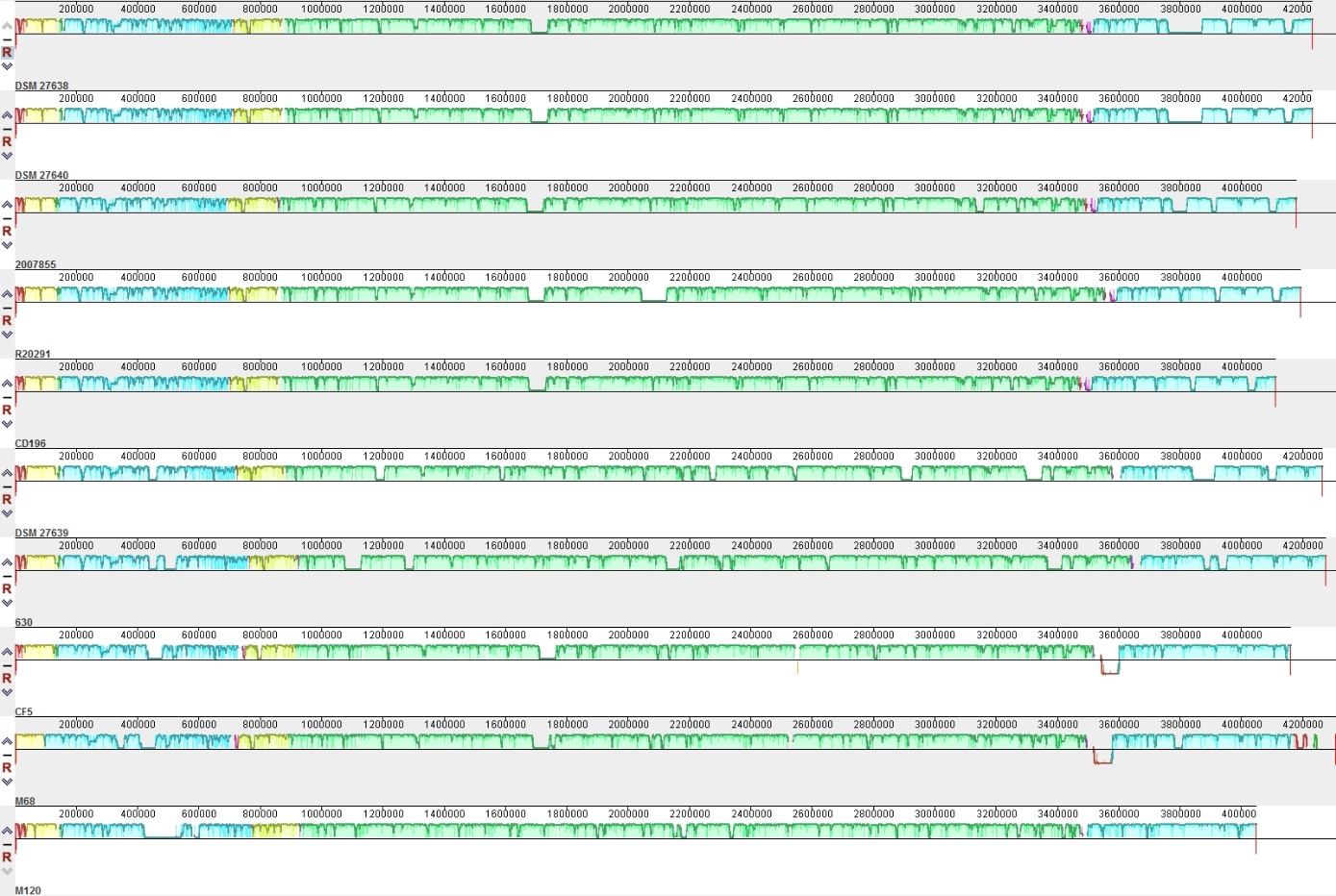

Supplement: Supplementary file 3 — Mauve whole genome alignments of ten linearized C. difficile strains from four different PCR ribotypes. In this alignment process locally collinear blocks (LCBs) were generated. Boxes with identical colors represent LCB, indicating homologous DNA regions shared between the chromosomes without sequence rearrangement. Lines collate aligned segments between genomes. The vertical bars denote the conservation level, and upward and downward orientations relative to the genome line indicates collinear and inverted regions, respectively. The only non syntenic region of the genomes is indicated by the red box. Sequences outside colored blocks do not have homologues in the other genome. (DOCX 419 kb) [file 12864_2017_4368_MOESM3_ESM.docx]

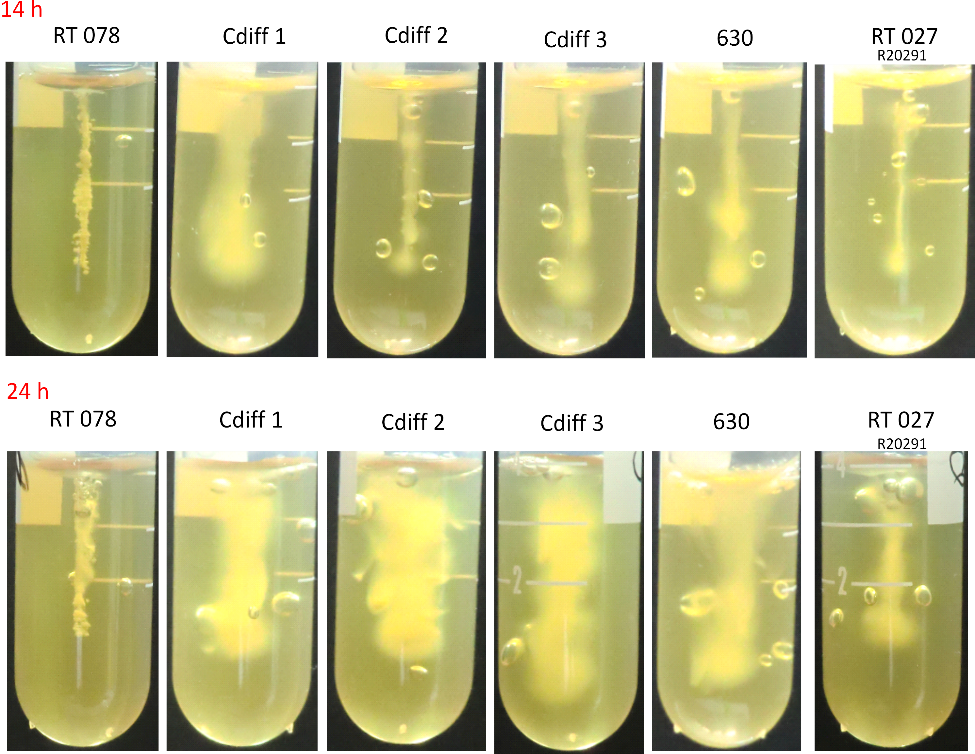

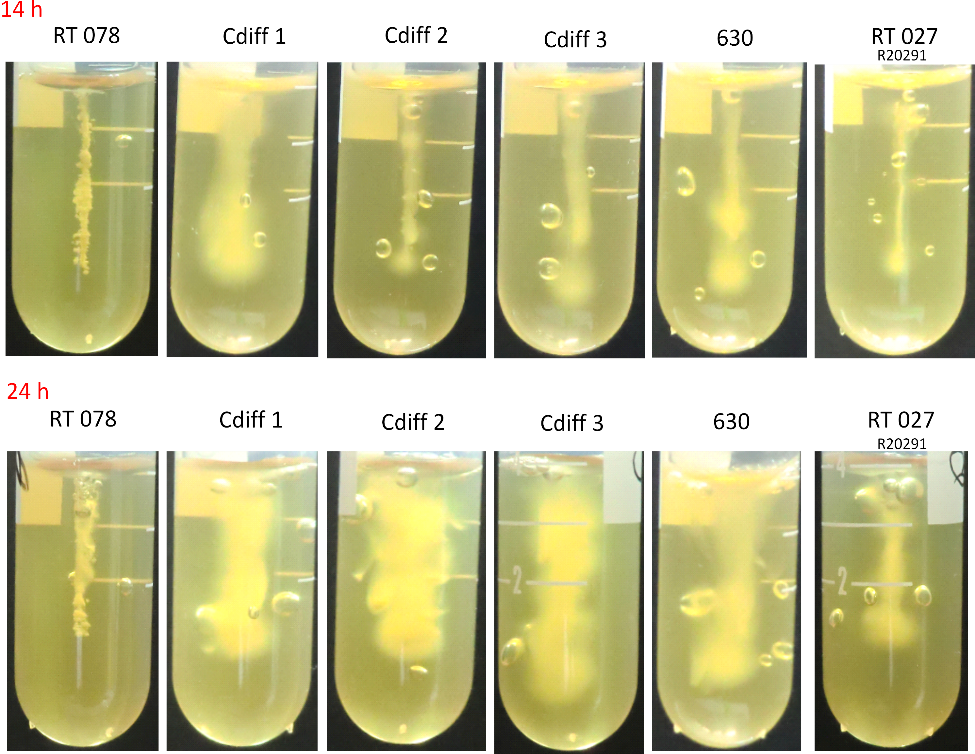

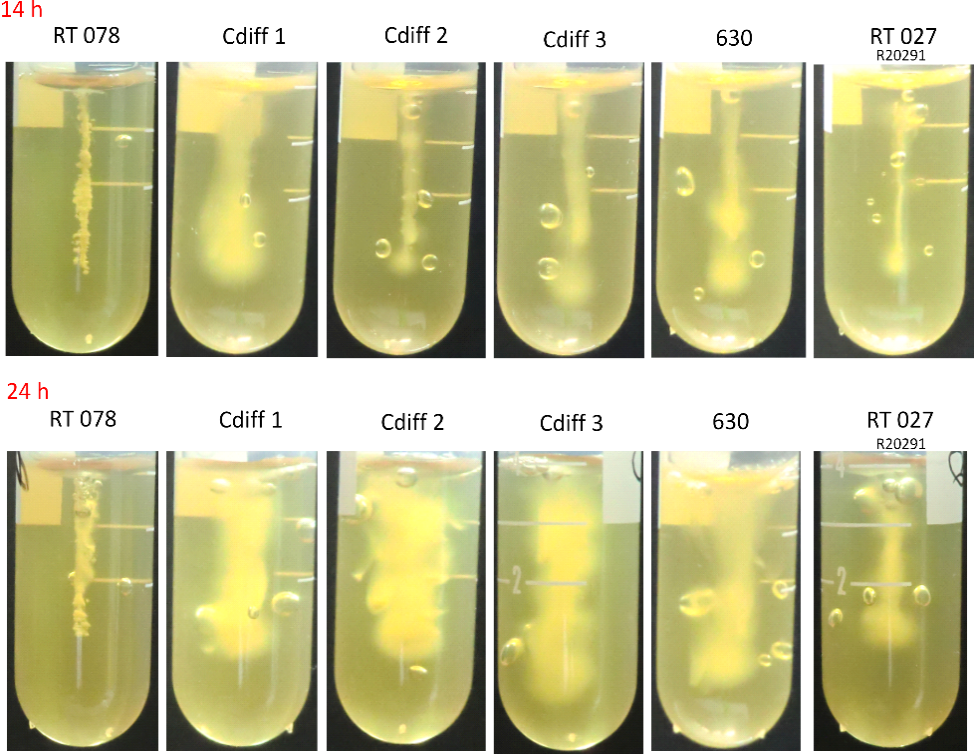

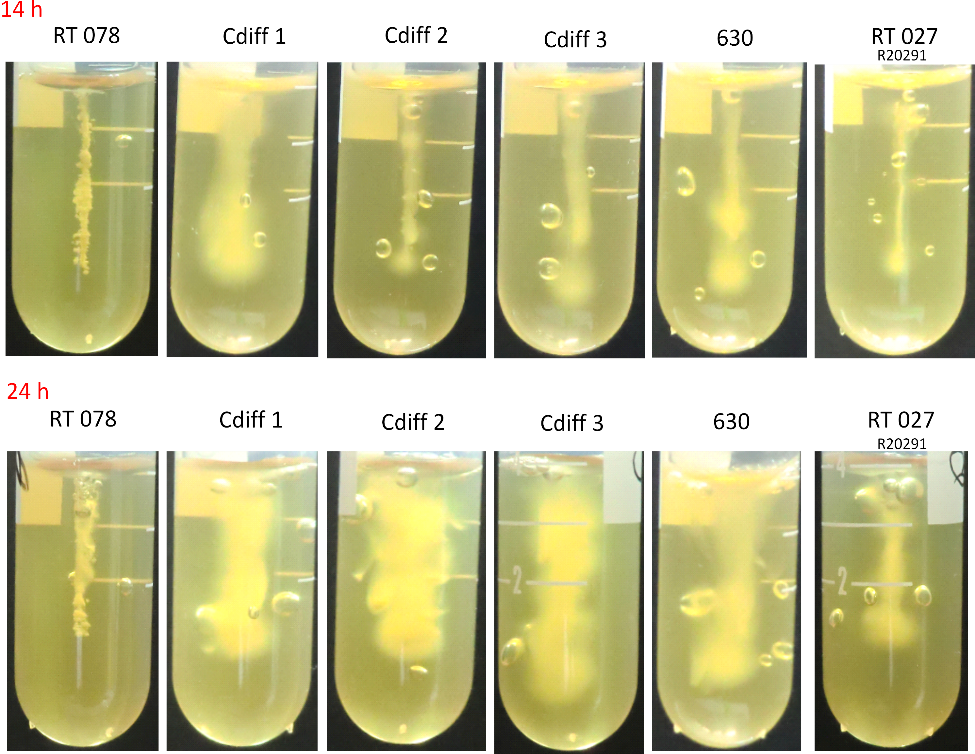

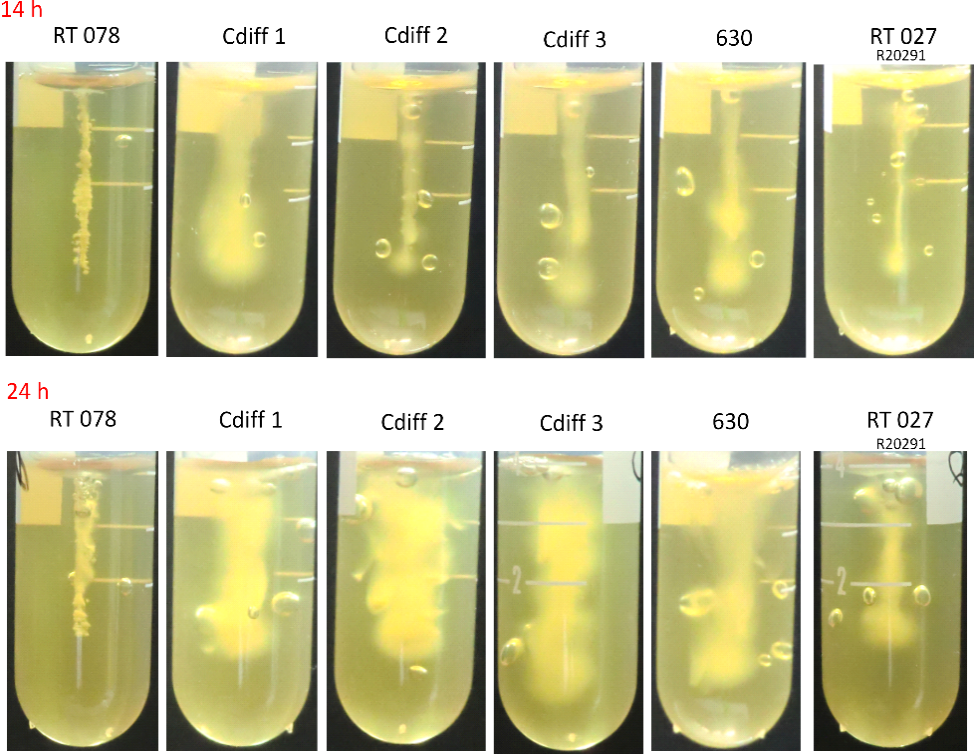

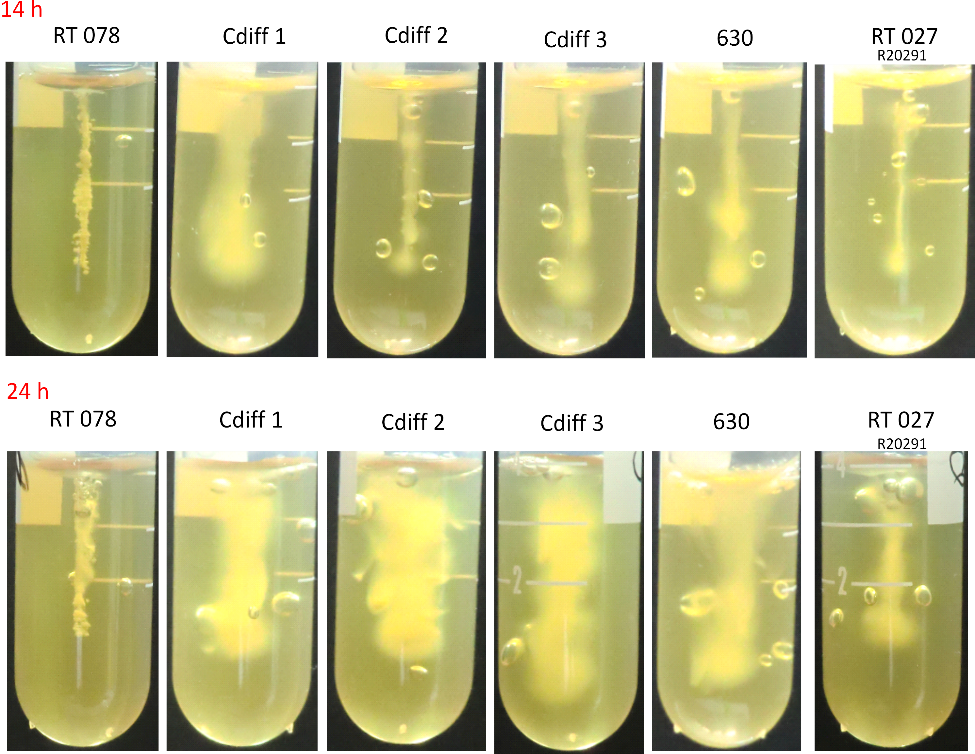


DSM 27638 DSM 27640 R20291 DSM 27639 630

**14h**

**24h**

RT 027

RT 012

RT 078

**A**

DSM 29747


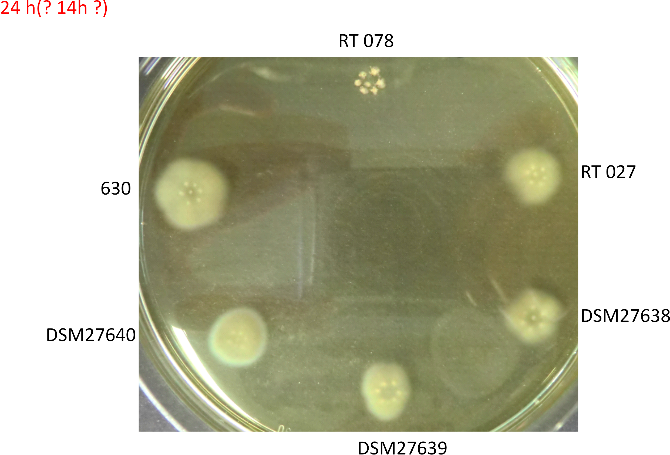


630

DSM 27639

DSM 29747

R20291

DSM 27638

DSM 27640

**B**

**14h**


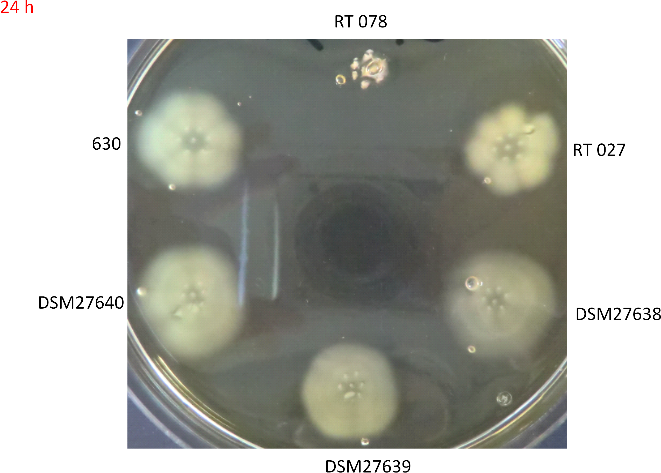


DSM 27638

DSM 27639

R20291

**24h**

DSM 29747

630

DSM 27640

Supplement: Supplementary file 7 — Motility assay of C. difficile isolates. RT 012 strains (630; DSM 27639), RT 027 strains (R20291; DSM 27640; DSM 27638) and non-motile RT 078 (DSM 29747) as a reference were analyzed on 0.3% BHI-agar. Following 1 to 2 days of post inoculation, the diffusion radius was monitored in semi-solid hungate tubes (A) and on semi-solid agar plates (B), respectively. Note, all strains produced gas. (DOCX 11038 kb) [file 12864_2017_4368_MOESM7_ESM.docx]
