# Supplementary material for: Comparative genome and phenotypic analysis of three Clostridioides difficile strains isolated from a single patient provide insight into multiple infection of C. difficile
Source: BMC Genomics. 2018 Jan 2;19:1. doi: 10.1186/s12864-017-4368-0 (PMC5749029; doi:10.1186/s12864-017-4368-0)

DSM 27638 – veg. cells and spores

DSM 27638 – spores

DSM 27639 – veg. cells and spores

DSM 27639 – spores

DSM 27640 – veg. cells and spores

DSM 27640 – spores


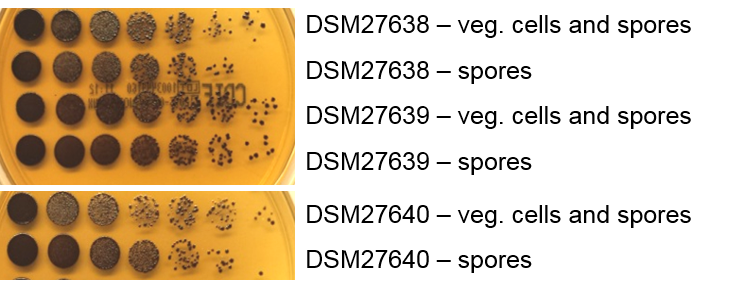

Supplement: Supplementary file 2 — Sporulation assay of DSM 27638, DSM 27639 and DSM 27640 on ChromID plates after 5 days incubation on BHIS. All three isolates show a comparable count of spores that germinated on the plate. (DOCX 275 kb) [file 12864_2017_4368_MOESM2_ESM.docx]
