# Supplementary material for: Comparative genome and phenotypic analysis of three Clostridioides difficile strains isolated from a single patient provide insight into multiple infection of C. difficile
Source: BMC Genomics. 2018 Jan 2;19:1. doi: 10.1186/s12864-017-4368-0 (PMC5749029; doi:10.1186/s12864-017-4368-0)
Supplement: Supplementary file 4 — Genes assigned to resistance. (DOCX 16 kb) [file 12864_2017_4368_MOESM4_ESM.docx]

**Table S1: Genes assigned to resistance.**

| **Annotation** | **gene** | **DSM 27638** | **DSM 27639** | **DSM 27640** |
| --- | --- | --- | --- | --- |
| tetracycline resistance protein |  | CDIF27638_00125 | CDIF27639_00118 | CDIF27640_00125 |
| antibiotic resistance ABC transporter ATP-binding protein |  | CDIF27638_00536 | CDIF27639_00550 | CDIF27640_00536 |
| quaternary ammonium compound-resistance protein | *sugE* | CDIF27638_00574 | CDIF27639_00583 | CDIF27640_00574 |
| teicoplanin resistance protein | *vanZ* | CDIF27638_01250 | CDIF27639_01261 | CDIF27640_01250 |
| vancomycin b-type resistance protein VanW | *vanW* | CDIF27638_01461 | CDIF27639_01470 | CDIF27640_01460 |
| vancomycin/teicoplanin A-type resistance protein | *vanG* | CDIF27638_01732 | CDIF27639_01691 | CDIF27640_01731 |
| tellurium resistance protein |  | CDIF27638_01740 | CDIF27639_01699 | CDIF27640_01739 |
| tellurium resistance protein |  | CDIF27638_01741 | CDIF27639_01700 | CDIF27640_01740 |
| tellurium resistance protein |  | CDIF27638_01742 | CDIF27639_01701 | CDIF27640_01741 |
| tellurium resistance protein |  | CDIF27638_01744 | CDIF27639_01703 | CDIF27640_01743 |
| tellurite resistance protein |  | CDIF27638_01745 | CDIF27639_01704 | CDIF27640_01744 |
| tellurium resistance protein |  | CDIF27638_01758 | CDIF27639_01717 | CDIF27640_01757 |
| tellurium resistance protein |  | CDIF27638_01919 | CDIF27639_01885 | CDIF27640_01918 |
| tellurium resistance protein |  | CDIF27638_01920 | CDIF27639_01886 | CDIF27640_01919 |
| multi-drug resistance efflux pump |  | CDIF27638_01930 | CDIF27639_01896 | CDIF27640_01929 |
| glyoxalase/bleomycin resistance protein/dioxygenase |  | CDIF27638_02022 | CDIF27639_01998 | CDIF27640_02021 |
| vancomycin b-type resistance protein VanW |  | CDIF27638_02236 | CDIF27639_02249 | CDIF27640_02235 |
| toxic anion resistance protein |  | CDIF27638_02414 | CDIF27639_02444 | CDIF27640_02413 |
| antibiotic resistance ABC transporter ATP-binding protein |  | CDIF27638_02681 | CDIF27639_02714 | CDIF27640_02680 |
| multidrug resistance protein | *cme* | CDIF27638_03297 | CDIF27639_03406 | CDIF27640_03297 |
| nogalamycin resistance protein | *snorO* | CDIF27638_03318 | CDIF27639_03427 | CDIF27640_03318 |
| multidrug resistance protein |  | CDIF27638_03817 | CDIF27639_03904 | CDIF27640_03817 |
| glyoxalase/bleomycin resistance protein/dioxygenase |  | CDIF27638_03824 | CDIF27639_02732 | CDIF27640_03824 |
