# Supplementary material for: Comparative genome and phenotypic analysis of three Clostridioides difficile strains isolated from a single patient provide insight into multiple infection of C. difficile
Source: BMC Genomics. 2018 Jan 2;19:1. doi: 10.1186/s12864-017-4368-0 (PMC5749029; doi:10.1186/s12864-017-4368-0)
Supplement: Supplementary file 5 — DNA gyrase subunit A alignment. The resistance phenotype is encoded by the amino acid substitution at the position 82 of GyrA. (DOCX 33 kb) [file 12864_2017_4368_MOESM5_ESM.docx]

70 75 80 **82** 85 90

RT 012

RT 017

RT 078

RT 027

**M120** RIVGDVLGKYHPHGDTAVYYAMVR

**630** RIVGDVLGKYHPHGDTAVYYAMVR

**DSM 27639** RIVGDVLGKYHPHGDTAVYYAMVR

**M68** RIVGDVLGKYHPHGDTAVYYAMVR

**CF5** RIVGDVLGKYHPHGDTAVYYAMVR

**CD196** RIVGDVLGKYHPHGDTAVYYAMVR

**DSM 27640** RIVGDVLGKYHPHGD**I**AVYYAMVR

**2007855** RIVGDVLGKYHPHGD**I**AVYYAMVR

**DSM 27638** RIVGDVLGKYHPHGD**I**AVYYAMVR

**R20291** RIVGDVLGKYHPHGD**I**AVYYAMVR

*************** ********

#
