# Supplementary material for: Comparative genome and phenotypic analysis of three Clostridioides difficile strains isolated from a single patient provide insight into multiple infection of C. difficile
Source: BMC Genomics. 2018 Jan 2;19:1. doi: 10.1186/s12864-017-4368-0 (PMC5749029; doi:10.1186/s12864-017-4368-0)
Supplement: Supplementary file 5 [file 12864_2017_4368_MOESM6_ESM.docx]

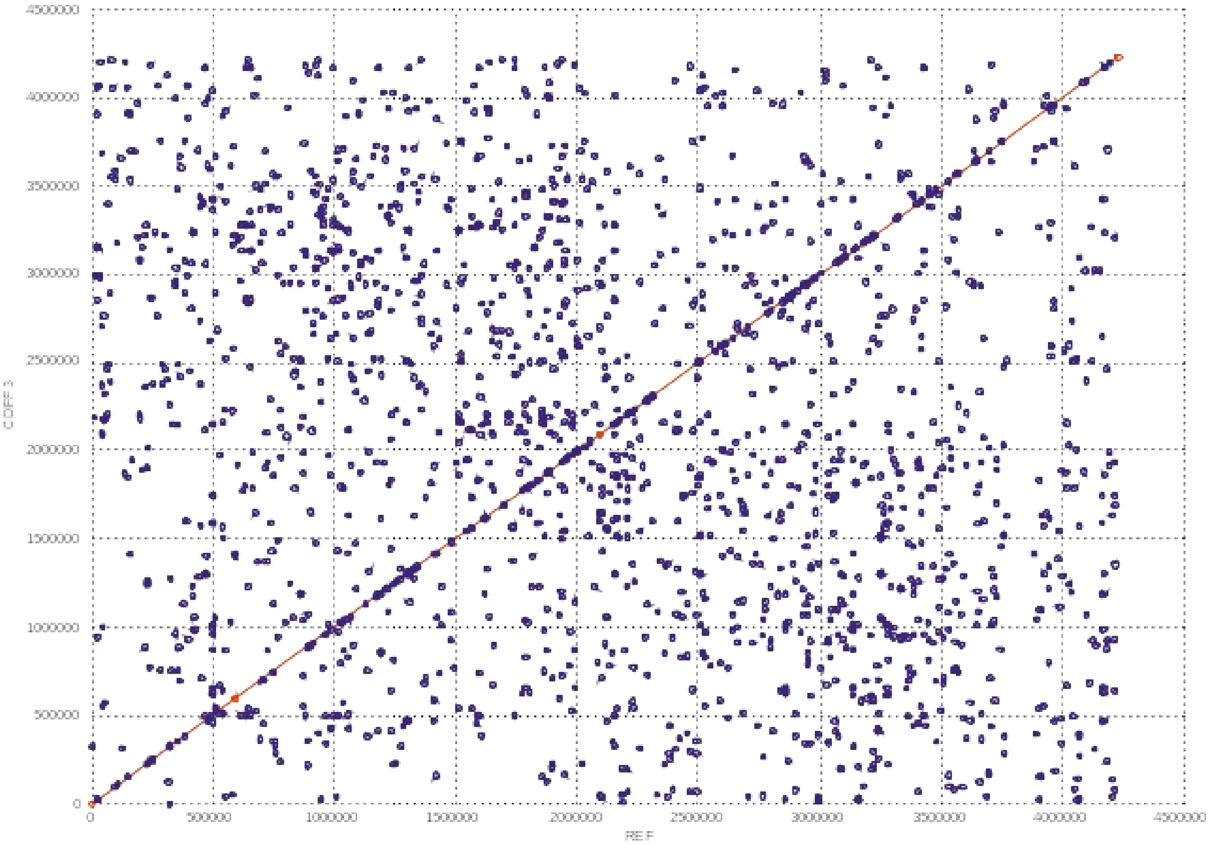

4500000
4000000
3500000
3000000
2500000
2000000
1500000
1000000
500000
0
CDIFF 3
0
500000
1000000
1500000
2000000
2500000
3000000
3500000
4000000
4500000
REF
